# COVID-19 Vaccination in Patients with Cancer

**DOI:** 10.3390/cancers14102556

**Published:** 2022-05-23

**Authors:** Hitomi Suzuki, Tomohiro Akiyama, Nobuko Ueda, Satoko Matsumura, Miki Mori, Masatoshi Namiki, Norikazu Yamada, Chika Tsutsumi, Satoshi Tozaki, Hisayuki Iwamoto, Shun Torii, Yuichiro Okubo, Kiyosuke Ishiguro

**Affiliations:** 1 Department of Breast and Endocrinology Surgery, Ichinomiya Nishi Hospital, Ichinomiya 494-0001, Japan; nyatomiki@yahoo.co.jp (M.M.); sumes1969@yahoo.co.jp (M.N.); kk.sm-fs.ed-fp.21@softbank.ne.jp (N.Y.); chikatsutsumi3322@gmail.com (C.T.); tozaki.3104.0811@gmail.com (S.T.); iwamoto0820@outlook.jp (H.I.); tsncu_m@yahoo.co.jp (S.T.); yukubo0615@gmail.com (Y.O.); k-ishiguro@anzu.or.jp (K.I.); 2 Advanced Research Laboratories, Tokyo City University, Tokyo 158-0082, Japan; 3 Graduate School of Education, Kyoto University, Kyoto 606-8501, Japan; 4 Graduate School of Global Environmental Studies, Sophia University, Tokyo 102-8554, Japan; 5 School of Integrative and Global Majors, University of Tsukuba, Tsukuba 305-8577, Japan; 6 Graduate School of Information Technology, Kobe Institute of Computing, Kobe 650-0001, Japan; 7 School of Social and Behavioral Sciences, Nanjing University, Nanjing 210023, China; 8 Institute for Humanities in Africa, University of Cape Town, Rondebosch 7701, South Africa; 9 Peer Ring Association, Yokohama 224-0001, Japan; ueda@peer-ring.com (N.U.); msmscco@gmail.com (S.M.)

**Keywords:** cancer, COVID-19, vaccine, online questionnaire, doctor–patient communication

## Abstract

**Simple Summary:**

Patients with cancer are concerned about the effects of the COVID-19 vaccination while authorities search for ways to encourage vaccination; however, many points are still unclear. To remedy this situation, we conducted an online survey of 1182 female patients with cancer. The results showed that 768 were concerned about the vaccine, and 726 consulted with their attending physicians about the vaccination. The results also showed significantly higher vaccination rates among the patients who had consulted with their physicians. We found that consulting with attending physicians appeared to be linked to reduced anxiety, decisions about the timing of the vaccination, and higher vaccination rates. This was corroborated by the result of the cross-analysis of vaccination status and information sources about the vaccination. Therefore, we concluded that consulting with a physician about vaccination alleviates the concerns of patients with cancer and encourages them to get vaccinated.

**Abstract:**

Patients with cancer are concerned about the effects of the COVID-19 vaccination. We conducted an online survey on the COVID-19 vaccination status and side effects among patients with cancer in Japan between 8 and 14 August 2021. We included 1182 female patients with cancer aged 20–70 years and registered on an online patient website. Of the patients, 944 had breast cancer, 216 had gynecological cancer, 798 were undergoing drug/radiation therapy, and 370 were in follow-up. At the time of the survey, 885 patients had already received at least one dose. Of these, 580 had also received their second dose. The incidence rate of side effects was equivalent to previous reports. In patients with breast cancer, problems such as the onset or worsening of lymphedema or axillary lymphadenopathy metastasis requiring differential diagnosis were encountered following vaccination. A total of 768 patients were concerned about the vaccine at some point, and 726 consulted with their attending physicians about the timing or side effects of the vaccination. Of the 110 patients undergoing chemotherapy or radiation therapy, 75 adjusted the timing of the vaccination based on their therapy. The cross-analysis revealed that 81% of those who consulted their physician had received at least one dose of the COVID-19 vaccination compared with 65% of those who had not consulted their physician. Consulting with a physician about the COVID-19 vaccination was found to alleviate the concerns of patients with cancer and encourage them to get vaccinated.

## 1. Introduction

The coronavirus disease 2019 (COVID-19) spread throughout the world from Wuhan, China, and various situations of emergency were declared in Japan. There have been second, third, and fourth waves of the COVID-19 pandemic, with no end in sight. The COVID-19 pandemic has been an unprecedented challenge not only for the general public but also for clinicians and patients. The postponement of cancer treatment reducing its therapeutic effects and curability is a concern [1,2]. Therefore, performing cancer treatment without delay has been one of the main issues in the medical field during the pandemic.

COVID-19 vaccination has been developed as a means of preventing pandemics. The two vaccines that received approval for use in Japan were the BNT162b2 mRNA COVID-19 vaccine (Pfizer-BioNTech vaccine) and the SARS-CoV-2 mRNA-1273 vaccine (Moderna vaccine). Currently, these two vaccines are widely utilized around the world. The elderly, healthcare professionals, and people with underlying health conditions, such as cancer, were the first to get vaccinated against COVID-19 in Japan. Subsequently, other countries have gradually caught up to this vaccination campaign.

While authorities search for ways to encourage vaccination, it is expected that patients who are receiving, or have recently received, cancer treatment may harbor worries about both the risk of infection and the vaccination itself. It has become well known that the secondary immune response to COVID-19 vaccination causes various side effects, including lymphadenopathy [3,4]. However, many points are still unclear regarding COVID-19 vaccination of patients with cancer, including the worries patients have and the potential side effects. To remedy this situation, we conducted an online survey of female patients with cancer about their COVID-19 vaccination status and attitudes toward vaccination.

## 2. Materials and Methods

The participants in this study were women with cancer registered with the online support group, Female-Specific Cancer Patient Support Community Peer Ring [5] which is open to international residents. This is an online community for women diagnosed with breast cancer, ovarian cancer, or cervical cancer. The group’s focus is peer support via social media. There are currently about 10,000 members, and the site is widely used by patients unable to participate in support groups in person due to side effects from treatment or location. Numerous surveys are conducted via the group’s app.

The third and fourth authors of the present study who are responsible persons of the Peer Ring Association used the mailing list as well as the social media of this community to conduct “Factual Investigation into the Impact of COVID-19 on Cancer Treatment”. We have previously conducted two investigations into the impact of COVID-19 on cancer treatment, investigating and reporting on matters such as the impact of COVID-19 on hospital visits and treatment after cancer diagnosis, changes in daily life or working status, participation in regular cancer screening, self-reported changes in health, and the incidence of depression and anxiety disorders [1]. This third online survey was on the topic of COVID-19 vaccination after cancer diagnosis and took place between 8 August 2021, and 14 August 2021. We received responses from 1182 participants. All the respondents were Japanese though it was not an inclusion criterion.

This study has been approved by the Ethics Review Board of Ichinomiya Nishi Hospital. The data collected in the surveys were anonymized so as not to include any personal information and the participating patients agreed when they replied to the purpose of this study and to the disclosure of the results.

## 3. Results

The majority of respondents were in their 40s or 50s. The age distribution, living situation, cancer types, stage of cancer, and current treatment of our sample are all shown in Table 1.

**Figure 1:**
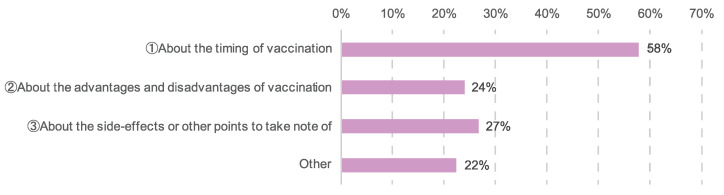
Content of discussions about COVID-19 vaccination with attending physicians. The number of responses was 726.

A total of 726 (61%) participants consulted with their attending physicians about COVID-19 vaccination. Of those, 420 (58%) discussed the timing of vaccination, 173 (24%) discussed the pros and cons of vaccination, 193 (27%) discussed the side effects and points of caution, and 163 (22%) discussed other matters, such as the injection site or underlying conditions besides cancer (Figure 1). Furthermore, 21 (3%) of the patients who consulted with their physicians were instructed to refrain from vaccination for reasons such as a history of allergies or decreased neutrophil levels due to ongoing chemotherapy.

**Figure 2:**
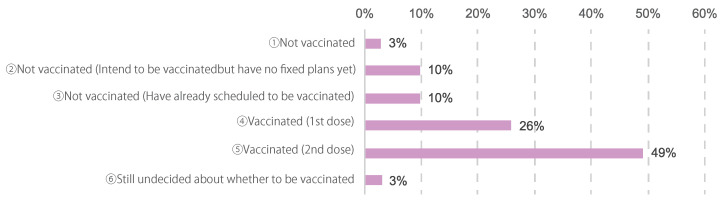
COVID-19 vaccination status. The number of responses was 1182.

The vaccination status of the participants was as follows: 34 (3%) had no intention of being vaccinated, 114 (10%) intended to be vaccinated but had not yet made plans, 114 (10%) had scheduled vaccinations, 305 (26%) had already received their first dose, 580 (49%) had already received their second dose, and 35 (3%) had not yet decided whether to be vaccinated (Figure 2).

**Figure 3:**
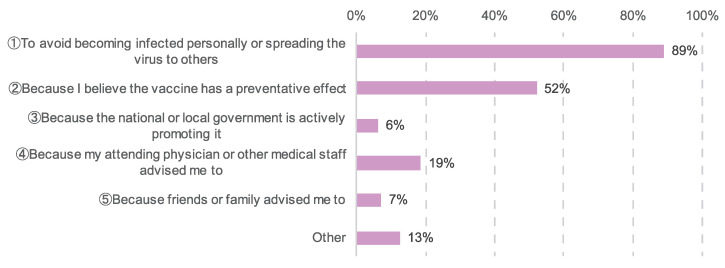
Reasons for vaccination among vaccinated patients and patients with plans to be vaccinated. The number of responses was 1113.

The participants who did not intend to be vaccinated gave the following reasons: “A history of allergies”, “Concerns about delayed cancer treatment due to side effects” and “Safety concerns about the vaccine”. Patients who planned to be vaccinated or had already been vaccinated gave the following as reasons for vaccination: “To prevent myself or others from being infected” (990 patients, 89%), “Because I believe the vaccines are effective in preventing the spread of the COVID-19 infection” (580 patients, 52%), “Because my attending physician or other medical staff suggested I take the vaccine” (208 patients, 19%), “Because friends or family suggested I take the vaccine” (78 patients, 7%), and “Because the national and local governments are actively promoting it” (68 patients, 6%) (Figure 3).

**Figure 4:**
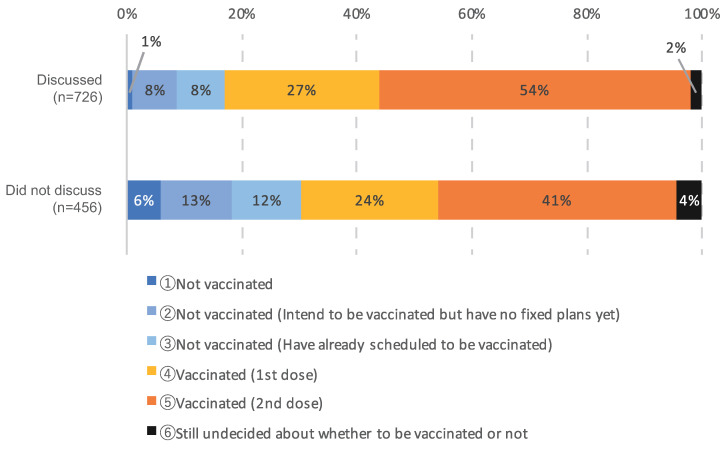
Cross-analysis of COVID-19 vaccination status and whether patients consulted with their attending physicians. The number of responses was 1182.

Cross-analysis of vaccination and treatment statuses and whether patients consulted with their attending physicians showed that 81% of patients who consulted with their attending physician had received at least one dose, whereas 65% of patients who had not consulted with their attending physician had received at least one dose (Figure 4).

**Figure 5:**
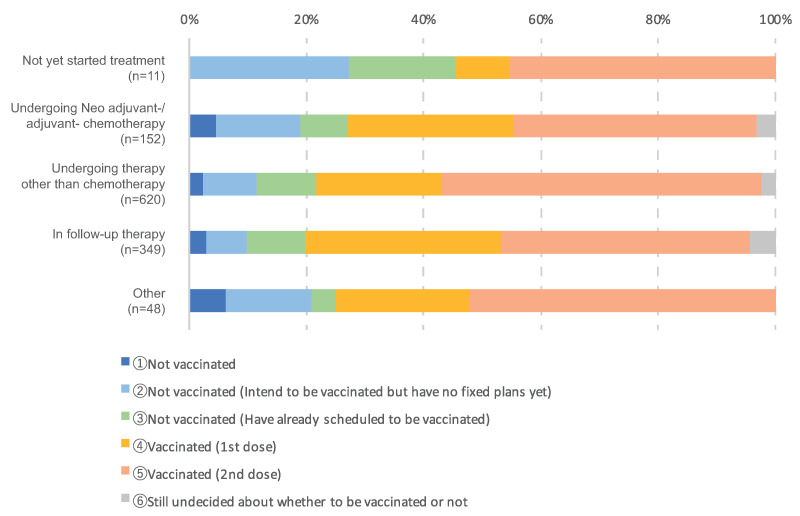
Cross-analysis of COVID-19 vaccination status and treatment status.

Figure 5 shows the result of the cross-analysis of COVID-19 vaccination status and treatment status. We found that 118 (78%) of patients were vaccinated or scheduled to be vaccinated during chemotherapy, and 553 (86%) were vaccinated or scheduled to be vaccinated during treatment other than chemotherapy including radiation therapy, and 300 patients (86%) were vaccinated or scheduled to be vaccinated during follow-up.

**Figure 6:**
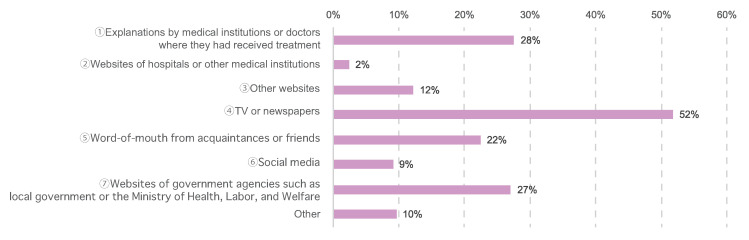
Information source about COVID-19 vaccination. The number of responses was 1182.

To acquire information about vaccination, 326 participants (28%) consulted medical institutions or doctors from whom they were receiving treatment, 29 (2%) consulted websites of hospitals or other medical institutions, 144 (12%) consulted other websites, 612 (52%) attained information from TV or newspapers, 265 (22%) attained information via word-of-mouth through acquaintances or friends, 108 (9%) attained information via social media, 320 (27%) consulted websites of government agencies such as local government or the Ministry of Health, Labor, and Welfare, and 113 (10%) consulted other sources (Figure 6).

**Figure 7:**
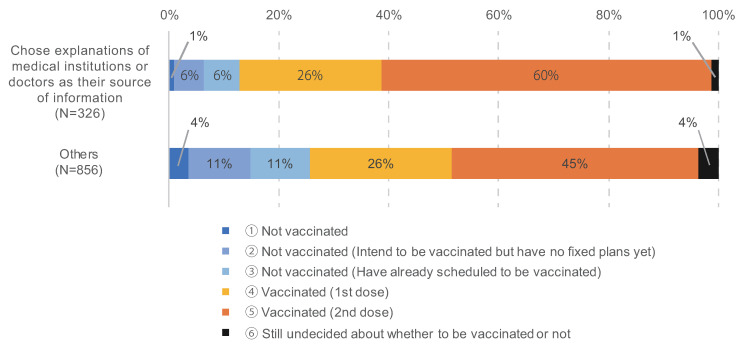
Cross-analysis of COVID-19 vaccination status and information source about COVID-19 vaccination. The number of respondents of “vaccinated or scheduled to be vaccinated” and “not vaccinated” are 999 and 183, respectively.

Figure 7 shows the result of the cross-analysis of COVID-19 vaccination status and information source about COVID-19 vaccination. It shows those who received information about COVID-19 vaccination from medical institutions or doctors where they had received treatment tended to be vaccinated though TV and newspapers are popular information source among the respondents. There was a significant difference in the χ-square test on whether or not to receive the COVID-19 vaccine between those who received information from treated medical institutions and doctors and those who did not (*p* < 0.001).

Among the 110 patients undergoing chemotherapy/radiation therapy, 75 had adjusted the timing of their vaccination based on their treatment schedule. Specifically, schedule adjustments were made based on the possibility of fever or bone marrow suppression, such as leaving a gap of a few days or a week before and after scheduled chemotherapy or arranging to be vaccinated after radiation therapy (Table 2).

**Figure 8:**
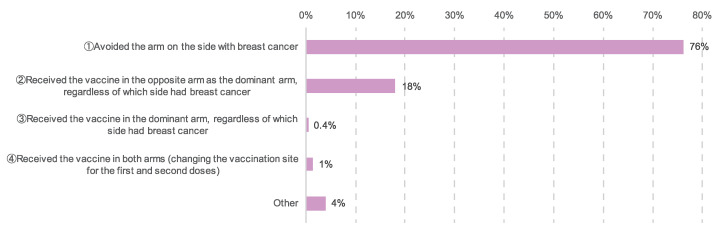
Inoculation sites. The number of responses was 709.

**Figure 9:**
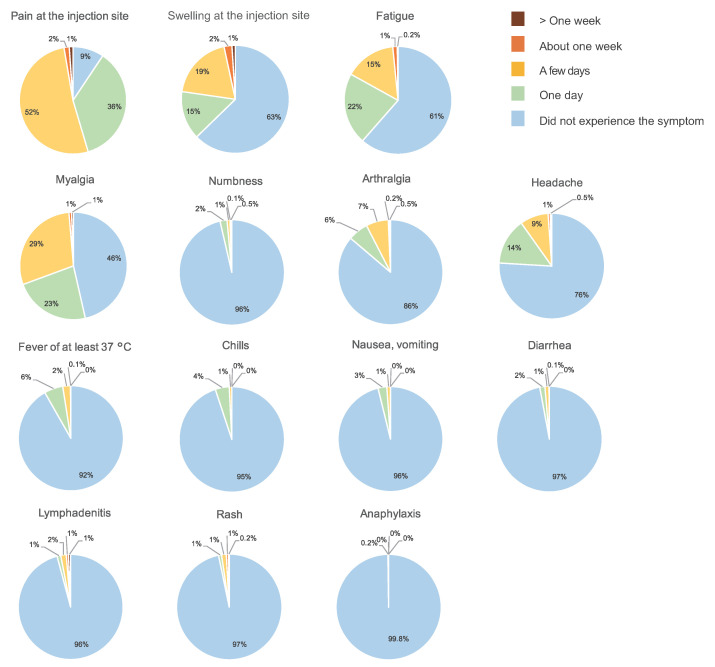
Side-effects from the first dose by time since first inoculation. The number of responses was 885.

**Figure 10:**
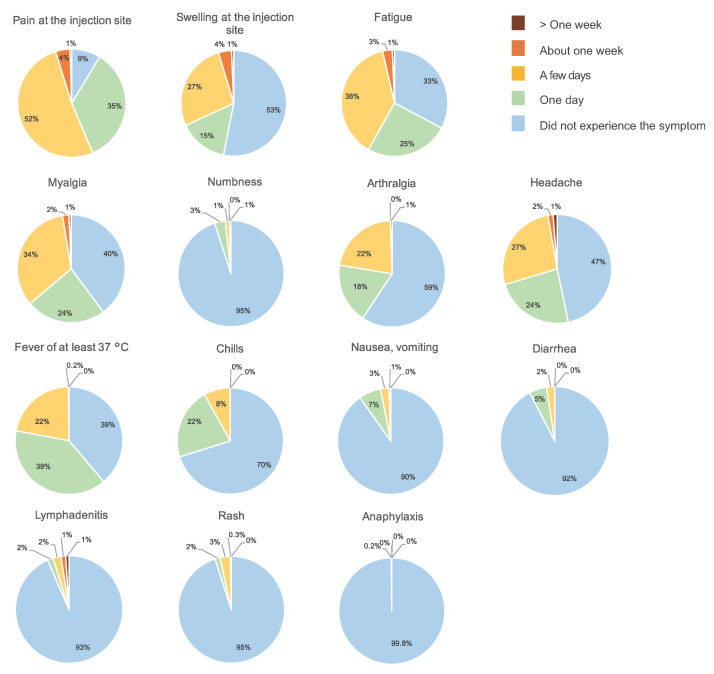
Side-effects from the second dose by time since second inoculation. The number of responses was 580.

Among the patients with breast cancer, 540 (76%) avoided receiving the vaccination in the arm on the side of the breast cancer, 128 (18%) received the injection in their nondominant arm, regardless of which side the cancer was, three (0.4%) received the injection in their dominant arm regardless of which side the cancer was, 10 (1%) changed sides between the first and second doses, receiving injections in both arms, whereas 28 (4%) received the injection somewhere other than their upper arm, such as in the thigh (Figure 8). The patients who had been vaccinated or had plans to be vaccinated were administered vaccines made by Pfizer (783 patients, 78%), Moderna (204 patients, 20%), and AstraZeneca (one patient, 0.1%). In 11 patients (1%), it was unclear which vaccine they received or planned to receive. Side effects experienced included fever, swelling at the injection site, and fatigue. There was a general tendency toward more side effects after the second dose (Figure 9 and Figure 10).

There were multiple reported instances of patients becoming worried that side effects of the vaccination such as prolonged bone pain or headaches might be signs of metastasis. In the breast patients with cancer, there were also cases with prolonged axillary lymphadenopathy that had to be screened to differentiate it from lymph node metastasis, as well as cases where the onset or worsening of lymphedema was observed, requiring additional treatment (Table 3).

**Figure 11:**
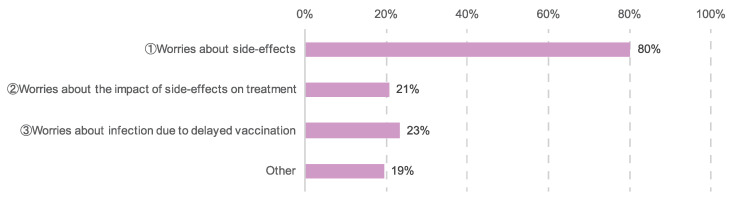
Concerns related to vaccination. The number of responses was 768.

A total of 768 (65%) participants reported concerns related to vaccination. In addition, 616 (80%) were concerned about side effects, 159 (21%) were concerned about the impact of side effects on cancer treatment, 179 (23%) were concerned about the spread of COVID-19 due to delayed vaccination, and 149 (19%) reported other concerns (Figure 11). Among other concerns, worries about the worsening of lymphedema among patients with breast cancer and about the safety of the vaccination or the long-term impact on the body were prevalent.

The survey also included an open-ended question about what participants would like to know about vaccination. Besides answers pertaining to more general topics such as “the effectiveness of the vaccines against mutant strains” or “the future spread of infection”, there were also many cancer-specific responses such as “data about the frequency of cases, death rates, and severe case rates among patients with cancer”, “data about the effectiveness of the vaccines in patients with cancer”, and “about the impact of the vaccines on lymphedema”.

## 4. Discussion

The spread of COVID-19 has already affected cancer treatment worldwide and, in these unprecedented circumstances, anxiety about COVID-19 among patients with cancer is extremely high. In a previous study, we found that over 90% of patients were anxious about catching the virus, and this anxiety was affecting their actual cancer treatment [1]. Other studies have reported fears of decreased survival rates among patients with cancer associated with the COVID-19 pandemic [6], whereas others have reported severe COVID-19 cases among cancer patients [7]. With COVID-19 vaccination programs becoming widely available, take-up of the vaccination should be encouraged even among patients with cancer undergoing treatment. Several reports showed benefits of COVID-19 vaccines for patients with cancer [8,9]. However, there is a deficiency of information about COVID-19 vaccinations and cancer.

### 4.1. Vaccination Status

Our survey also gathered data on participants’ vaccination status. Of those surveyed, 75% had received at least one dose and 49% had received two doses (Figure 2). Most of the patients had vaccinated to prevent themselves or others from being infected (Figure 3). This indicated that the vaccination rate of women diagnosed with cancer was higher than that of the domestic adult population.

The results showed that 61% of patients had consulted their attending physician about the COVID-19 vaccination (Figure 1). Cross-analysis of whether participants had consulted with their physicians and their vaccination status showed significantly higher vaccination rates among patients with cancer who had spoken to their physicians than those who had not (Figure 4). Direct conversation with attending physicians about the timing of the vaccination and its advantages and disadvantages appeared to be linked to reduced anxiety and decisions about the timing of the vaccination, as well as higher vaccination rates.

This is corroborated by the result of the cross-analysis of COVID-19 vaccination status and information source about COVID-19 vaccination which showed those who received information about COVID-19 vaccination from medical institutions or doctors where they had received treatment tended to be vaccinated (Figure 7). Most of the information from television and newspapers is general about the COVID-19 vaccine, and there is little personalized information for cancer patients. Therefore, it is probable that the information from medical institutions or doctors where they had received treatment mainly influenced whether or not to receive the COVID-19 vaccine.

### 4.2. Vaccination Timing

In our study, the vaccination rate was higher even during chemotherapy or endocrine therapy, which was similar to that of the patients under follow-up (Figure 5). We found that 68% of patients undergoing chemotherapy or radiation therapy had timed their vaccinations so as to keep their treatment days and vaccination days separate and prevent overlap of treatment days and the period in which the patient expected to experience vaccination side effects (Table 2).

In Japan, the Japan Breast Cancer Society have developed recommendation for managing breast cancer patients based on the guidelines from Japan Surgical Society [2]. However, the guidelines about cancer treatment and vaccination are rare. The Japan Cancer Association, Japan Society of Clinical Oncology, and the Japanese Society of Medical Oncology have jointly published a Q&A on cancer treatment and vaccinations [10], which is also advocated by the National Comprehensive Cancer Network’s guidelines [11].

Although vaccination is recommended before or after surgery, the guidelines of the Royal College of Surgeons in the UK do not recommend postponement of planned surgery or changing surgery dates to accommodate vaccination. Since fever is a vaccination side-effect, which may last from one to two days up to a week, a few days to a week between the vaccination and surgery is sufficient for the purposes of distinguishing whether fever is surgery-related or a vaccination side-effect [12]. Ko et al. provided guidelines regarding the timing of the vaccine and breast cancer surgery [13]. They recommend scheduling the vaccine at least one week before surgery so that symptoms such as fever can be correctly attributed to side effects from the vaccine rather than surgery [13]. The optimal timing for surgery and COVID-19 vaccination is still under discussion.

Vaccination is considered a positive action even during cancer treatment, including radiation therapy [14]. However, there is currently no data about the best way for patients with cancer to time vaccinations around treatment. Discussing vaccination timing with the attending radiotherapist and, where possible, aiming to receive vaccinations during weekends, when patients have no radiation therapy, in order to avoid treatment during the period of possible fever immediately after vaccination, is preferable.

Various academic societies have advocated the safety of the COVID-19 vaccination when undergoing pharmacotherapy, including chemotherapy, as shown in Table 4 [11,15,16,17,18]. All suggest that vaccination is preferable in patients with no history of allergy to any of the components of the vaccine. Waissengrin et al. showed that the COVID-19 vaccination had generally been suggested to be safe in cancer patients who treated with immune checkpoint inhibitors [19].

### 4.3. Side Effects

Approximately 80% of our survey respondents who had or intended to receive vaccination had received the Pfizer vaccination. The frequency of postvaccination side effects such as fatigue and fever was higher after the second dose than the first (Figure 9 and Figure 10), though this has already been established in previous reports [20,21,22,23].

Both Pfizer’s BNT162b2 package leaflet and the Centers for Disease Control and Prevention (CDC) in the US recommend vaccination in the deltoid muscle [24,25]. Some previous reports showed lymphadenopathy as a side-effect when the deltoid muscle is used as the vaccination site [3,4,26,27]. In cases where surgery and radiation therapy involve the axillary lymph node, as in breast cancer, vaccination in the contralateral side of the deltoid muscle or thigh is recommended [28]. Our study identified patients who were left anxious due to suspected recurrence of cancer metastasis in the axillary lymph node due to lymph node swelling after upper arm vaccination (Table 3). The study from Israel where a massive vaccination program was started earlier than our country reported the analysis of patients who presented with lymphadenopathy and history about vaccination [29]. This report showed that lymph node cortical thickening was thinner five weeks after vaccination [29]. The Society of Breast Imaging suggests that, since axillary lymph node swelling after vaccination can be confused with cancerous metastasis, an interval of 4–6 weeks after vaccination should be allowed before imaging examinations such as CT scans [30]. A longer delay of breast screening and all non-urgent imaging exams (at least six weeks from vaccine) is also advocated by the radiology scientific expert panel [31]. Vaccination timing should be taken into consideration in patients with cancer as scheduling adjustments may lead to delays in treatment if the timing of examinations after diagnosis overlaps with vaccination.

It is not yet clear whether COVID-19 vaccination increases the risk of lymphedema, but there were respondents in this study who both developed lymphedema and who experienced worsening of existing lymphedema after vaccination (Table 3). Onset or exacerbation of lymphedema after vaccination should be taken into consideration, particularly in patients with a history of lymphedema. In our study, 76% of patients avoided receiving the vaccination in the arm on the side of the breast cancer (Figure 8). Altering the vaccination area to the arm contralateral to the affected side or the thigh is thought to be preferable in such cases. Patients at risk of lymphedema, those who have undergone axillary dissection, and those who have received radiation therapy should discuss the possibility of lymphedema as well as the timing and site of their vaccinations with their oncologist in advance.

### 4.4. Vaccination Anxiety and Information Literacy

In this study, 65% of the respondents reported experiencing anxiety about vaccination, revealing that many patients with cancer have concerns not just about COVID-19 infection but also about the vaccination (Figure 11). When respondents were asked how they had obtained information about vaccinations, 52% had relied on television and newspapers more than physicians and medical institutions, while others had relied on word-of-mouth and social media (Figure 6). There have been reports of mental health issues arising from excessive continued exposure to COVID-19 information on social media [32,33]. There is an impetus not just for medical professionals, but for society as a whole, to disseminate and utilize accurate medical information.

## 5. Conclusions

In this study, we were able to determine the COVID-19 vaccination status of patients with cancer and gain insight into the attitudes and feelings of this demographic regarding the vaccine. More people will be inoculated again in the third round of vaccinations, yet there is presently no prospect of bringing the infection under control, meaning an extension of the anxiety felt by patients with cancer. The results of this study indicate that it is necessary to establish support systems that can provide peace of mind to patients with cancer facing vaccination and cancer treatment.

However, there are some limitations of the present study. First, this study was limited by unclear patient demographics as five respondents’ user profiles were private. Second, as this study was conducted on social media, there may also be issues of authenticity. Future research will need to collect and analyze data from a more reliable source that provides clear information about patient demographics. Third, this survey was conducted among only 1182 female patients with cancer aged 20–70 years. No male patients were included as we focused on breast and gynecological cancers. Fourth, all the respondents were Japanese. This limitation was probably due to the fact that most parts of the website and social media of the Peer Ring Association are provided in Japanese language. Nevertheless, we included 1182 female patients of which 944 had breast cancer and 216 had gynecological cancer. Among them, there was a total of 221 new cancer patients, including 13 cases of before treatment, 153 cases of neoadjuvant/adjuvant chemotherapy, 20 cases of radiation therapy, and 35 cases of inserting expander in breast. In general, neoadjuvant/adjuvant chemotherapy, radiation therapy, and tissue-expander insertion for breast reconstruction are performed within one year after cancer diagnosis [34,35,36,37,38]. Thus, it is reasonable to think the 221 had been induced within a year though the questionnaire did not ask duration after cancer diagnosis. According to National Cancer Center in Japan, annual incidence of breast cancer was 93,858 among women and 661 among men, and that of gynecological cancer was 41,116 patients (10,978 for cervix uteri, 17,089 for corpus uteri, and 13,049 for ovary) in Japan in 2018. Thus, the 221 patients are equal to 0.164% of the annual female incidence of breast cancer and gynecological cancer. Since the present study has probably covered more than 1/1000 population in the both types, it may be possible to generalize to female patients of breast cancer and gynecological cancer in Japan. However, large-scale investigations with a broader perspective remain necessary.

## Figures and Tables

**Table 1:** Patient characteristics. The number of responses was 1182.

		No. of Patients (%)
**Age (years)**	20s	4 (0.3)
	30s	94 (8)
	40s	467 (40)
	50s	520 (44)
	60s	86 (7)
	70s	6 (1)
	No response	5 (0.4)
**Households **	Single-person	154 (13)
	Two or more	1028 (87)
**Type of cancer **	Breast cancer	944 (80)
	Cervical cancer	41 (3)
	Endometrial cancer	82 (7)
	Ovarian cancer	93 (8)
	Others	22 (2)
**Stage of cancer **	0 (DCIS)	71 (6)
	I	458 (39)
	II	403 (34)
	III	153 (13)
	IV	69 (6)
	Unknown	28 (2)
**Stage of treatment **	Before treatment	13 (1)
	Neoadjuvant chemotherapy	27 (2)
	Adjuvant chemotherapy	126 (11)
	Radiation therapy	20 (2)
	Endocrine therapy	625 (53)
	Inserting expander in breast	35 (3)
	Follow-up	370 (31)

**Table 2:** Specific adjustments made. The number of responses was 75.

Type of Adjustment	Details
Arranged to be vaccinated during the interval between administration of anticancer drugs	Received the vaccine right around the midpoint of the interval between the days of anti-cancer drug administration Attending physician scheduled the vaccination to be a week after anti-cancer drug administration After anti-cancer drug administration, avoiding the following week, received the vaccine the week after that, then anti-cancer drugs were administered the following week Chose the 3rd week after anti-cancer drug; selected the Pfizer vaccine to match the anti-cancer drug course
Changed the treatment schedule of chemotherapy because of vaccination	Spread out the interval of chemotherapy to 4 weeks, allowing at least one week before and after vaccination
Avoided being vaccinated when white blood cell count was lowered from anticancer drugs	Arranged to be vaccinated when immune function returned after anti-cancer drug administration Avoided overlapping with the period of myelosuppression
Avoided vaccination on the same day as molecular targeted drugs were administered	Allowed at least one week after the administration of molecular targeted therapy Was advised by chemotherapy doctors/nurses to avoid the three days before and after molecular targeted drug administration, and to arrange to be vaccinated early the next week after drug administration (administration every three weeks) Avoided the day of herceptin monotherapy, but received the vaccine (both 1st and 2nd doses) two days after treatment
Avoided administering anti-cancer drugs during the week of vaccination	Avoided administering anti-cancer drugs on the week of vaccination Allowed about one week after chemotherapy before vaccination
Since chemotherapy could not be paused, received the vaccine on a Friday, so it would work out even if there were side-effects	Since it was during radiation therapy, decided to be vaccinated on Friday, fearing a fever Received the vaccine after irradiation on Friday in order to be able to rest well for two days after vaccination
Received the vaccine on a different day from family	Taking anti-cancer drugs orally. Allowed one week from the next hospital visit (avoiding the possibility of becoming unable to see a doctor due to fever) and chose a different vaccination from family

**Table 3:** The worries caused by side-effects after vaccination, which were identified based on a free response question. The number of responses was 885.

Type of Worry	Responses from the Participants
Onset or worsening of lymphedema	Despite having already undergone lymph node dissection for breast cancer and receiving the first dose of the vaccine in the left arm, opposite the cancer side, I got lymphedema in my right arm, on the cancer side, two days later. I’m having a difficult time. I received the vaccine on the healthy side, but the lymphedema on the cancer side got worse. The swelling of the lymphedema that had been stable for the past few years got worse by the day, becoming numb the following week, and I suffered from a heavy feeling and pain. I had no choice but to receive conservative treatment. I currently sleep on my side, keeping the cancer side up, but since the pain after receiving the injection was strong I unintentionally ended up sleeping on the other side, causing the lymphedema to worsen.
Impediments to daily life and housework	Being unable to raise my arm up disrupted my daily activities. Since I had a 38.8 °C fever, I was unable to go out shopping, etc. After the second dose, I was bedridden and unable to do housework because of side-effects of muscle pain and chills.
Impact on work	I was told by my attending physician that I would be given the vaccine in the opposite arm from the operation, meaning that I was vaccinated in my dominant arm. Later, the pain in my shoulder was dreadful, severely impeding my ability to work. I left work early because of the side-effects, and my boss changed my shift for me.
Worries about impact on hospital visits or treatment	It means being imaged while the lymphedema is swollen. I’m worried that it will be mistaken for a relapse or metastasis. I had prolonged nausea and vomiting and was unable to take tamoxifen. Since I couldn’t take a break from the radiation therapy, even though I had a fever the day after receiving the vaccine, I still had to go to the hospital. It was quite difficult.
Impact on cancer treatment or surgery	Since I received the second dose about one week after the operation, it was scary because I didn’t know if the pain in my body and the fever were because of the operation or a side-effect of the vaccine. My neutrophil count decreased rapidly, so I had to suspend taking my medicine. It was difficult to tell the difference between the side-effects of the anti-cancer drugs and the vaccine.
Feeling worried	There was something like an odd feeling of worry, and I was unable to sleep. Since I took the vaccine on the same day as my family, I worried about how long the side-effects of my other family members would continue.
Worries about metastasis	I experienced extreme pain three days after the first dose in my left rib. Since the pain was like a broken bone, I was checked for bone metastasis, but there was nothing out of the ordinary. There is a lingering mild headache and nausea, and I worry about whether it is brain metastasis.

**Table 4:** Guidelines of academic societies on COVID-19 vaccination during cancer drug therapy.

Organization	Summary of the Guideline
National Comprehensive Cancer Network (NCCN) [11]	Most people with cancer should get the vaccines as soon as they can.
European Society for Medical Oncology (ESMO) [15]	Considering the data for vaccines other than for COVID-19, vaccine effectiveness and safety is expected to be similar to nonpatients with cancer. Effectiveness varies depending on individual circumstances, but the benefits of vaccination are expected to significantly outweigh the risks. Ideally, the vaccine should be taken before cancer treatment, but it is also acceptable to take it during treatment if treatment has already begun.
American Society of Clinical Oncology (ASCO) [16]	Patients receiving cancer treatment may also be vaccinated. To avoid reducing the effectiveness of the vaccine, vaccination in the interval between administration of anticancer drugs may be considered.
American Association for Cancer Research (AACR) [17]	It is recommended that patients receiving cytotoxic anticancer drug treatment and immunotherapy be vaccinated preferentially.
National Cancer Institute (NCI) [18]	Patients with cancer may also be vaccinated. However, the possibility that vaccine effectiveness will be reduced for patients in an immunosuppressive state cannot be rejected, so patients should continue to take sufficient precautions against infection even after vaccination.

## Data Availability

Original images and CSV files may be obtained from the corresponding author upon request.

## Abbreviations

The following abbreviations are used in this manuscript:

AACR	American Association for Cancer Research
ASCO	American Society of Clinical Oncology
COVID-19	Coronavirus Disease 2019
ESMO	European Society for Medical Oncology
NCCN	National Comprehensive Cancer Network
NCI	National Cancer Institute
